# Structure of the *Arabidopsis* receptor kinase SRF6 ectodomain determined from crystals obtained using the LRR crystallization screen

**DOI:** 10.1107/S2059798326005498

**Published:** 2026-06-12

**Authors:** Alberto Caregnato, Ulrich Hohmann, Michael Hothorn

**Affiliations:** a Structural Plant Biology Laboratory, Department of Plant Science University of Geneva 1211Geneva Switzerland; University of Queensland, Australia

**Keywords:** crystallization screens, leucine-rich repeats, ectodomains, membrane receptors, *Arabidopsis thaliana*, plant development

## Abstract

Plant-specific membrane receptor kinases with structurally diverse extracellular domains regulate key processes in plant growth, development, immunity and symbiosis. Structural studies of these glycoproteins are often hampered by the limited quantities in which they can be obtained. Here, we describe the leucine-rich repeat (LRR) crystallization screen, which has enabled the successful crystallization and structure determination of multiple receptor kinase ecto­domains, including ligand- and co-receptor-bound complexes. As an example, we report the 1.5 Å resolution crystal structure of the LRR domain of STRUBBELIG-RECEPTOR FAMILY 6 (SRF6) from *Arabidopsis thaliana*. The SRF6 ectodomain contains seven LRRs and a disulfide-bond-stabilized N-terminal capping domain but lacks the canonical C-terminal cap and the *N*-glycosylation pattern typically found in other family members. Previously reported protein–protein interactions between the SRF6 and SRF7 ectodomains and the receptor kinases BRI1, BRL1, BRL3, SERK3 and BIR1–BIR3 could not be confirmed by quantitative isothermal titration calorimetry and grating-coupled interferometry assays, suggesting that these structurally conserved LRR receptor kinases may have signalling functions outside the brassinosteroid pathway.

## Abstract 2

A crystallization screen that has enabled the structural analysis of various extracellular domains of plant membrane receptor kinases is described together with the structure of the LRR domain of the receptor SRF6.

## 1. Introduction

Plant genomes harbour a large family of unique membrane receptor kinases (RKs) with extracellular leucine-rich repeat (LRR) extracellular domains (Shiu & Bleecker, 2001 ▸; Zhang *et al.*, 2026 ▸). Members of this family include receptor kinases that bind small-molecule, peptide or protein ligands (Hohmann *et al.*, 2017 ▸; Zhang *et al.*, 2017 ▸; Moussu & Santiago, 2019 ▸), co-receptor kinases required for receptor activation (Brandt & Hothorn, 2016 ▸) and receptor pseudo-kinases that regulate the formation of active signalling complexes (Ma *et al.*, 2017 ▸; Hohmann, Nicolet *et al.*, 2018 ▸). The extracellular LRR domains of plant RKs are often stabilized by disulfide bridges and are extensively *N*-glycosylated (Di Matteo *et al.*, 2003 ▸; Hothorn *et al.*, 2011 ▸; She *et al.*, 2011 ▸; Jia *et al.*, 2024 ▸). Consequently, crystallographic studies on plant LRR-RKs largely rely on ectodomains obtained by secreted expression in baculovirus-infected insect cells (Hothorn *et al.*, 2011 ▸; She *et al.*, 2011 ▸). The resulting low protein yields, often further reduced by enzymatic deglycosylation prior to crystallization (Okuda *et al.*, 2020 ▸), can severely limit the amount of sample available for high-throughput crystallization screening.

Testing a large number of variables that may influence sample crystallization (McPherson, 2004 ▸) often relies on commercial crystallization screens covering conditions previously associated with crystallization success (Berry *et al.*, 2006 ▸) or derived from systematic approaches (Gorrec, 2015 ▸; Gorrec & Bellini, 2022 ▸). Here, we report the LRR crystallization screen, which has so far yielded a dozen different plant LRR-RK ectodomain structures with minimal sample requirements (as low as 50 µl of concentrated protein sample per project). As an example, we describe the crystallization and structure solution of the LRR-RK STRUBBELIG-RECEPTOR FAMILY 6 (SRF6).

SRF6 is part of a small protein family of plant receptor kinases. Its founding member STRUBBELIG (SUB/SRF9) was originally described in a genetic screen for mutants defective in ovule development (Schneitz *et al.*, 1997 ▸) and is involved in plant organ development and cell-wall signalling (Chevalier *et al.*, 2005 ▸; Kwak & Schiefelbein, 2007 ▸; Kwak *et al.*, 2005 ▸; Eyüboglu *et al.*, 2007 ▸; Chaudhary *et al.*, 2020 ▸, 2021 ▸). The kinase activity of SUB appears to be dispensable for signalling (Chevalier *et al.*, 2005 ▸). The interaction of SUB with other membrane-integral proteins has been reported (Fulton *et al.*, 2009 ▸; Vaddepalli *et al.*, 2014 ▸; Chen *et al.*, 2023 ▸), but a validated ligand or interaction partner for its extracellular LRR domain remains to be identified.

Mutations in the STRUBBELIG-RECEPTOR FAMILY members SRF3 result in altered immune responses (Alcázar *et al.*, 2010 ▸, 2014 ▸; Atanasov *et al.*, 2018 ▸; Duan *et al.*, 2024 ▸) and iron homeostasis (Platre *et al.*, 2022 ▸). For SRF6, functions in brassinosteroid (BR) signalling (Eyüboglu *et al.*, 2007 ▸; Smakowska-Luzan *et al.*, 2018 ▸) and in the perception of the cell-wall breakdown product trigalacturonic acid (Bhasin *et al.*, 2025 ▸) have been proposed. Here, we report the crystal structure of the SRF6 ectodomain refined at 1.5 Å resolution, and biochemically characterize whether the SRF6 or SRF7 extracellular domains can interact with selected BR signalling components (Nolan *et al.*, 2020 ▸). At the molecular level, BR signalling is mediated by the steroid receptor LRR-RKs BRI1, BRL1 and BRL3 (Li & Chory, 1997 ▸; Caño-Delgado *et al.*, 2004 ▸; Caregnato *et al.*, 2025 ▸), which upon sensing a BR ligand interact with the LRR co-receptor kinases SERK1–SERK4 (Li *et al.*, 2002 ▸; Nam & Li, 2002 ▸; Gou *et al.*, 2012 ▸; Hohmann, Santiago *et al.*, 2018 ▸). The SERK co-receptors may be kept from interacting with BR receptors by constitutively interacting with the LRR receptor pseudokinases BIR1–BIR4 (Imkampe *et al.*, 2017 ▸; Hohmann, Nicolet *et al.*, 2018 ▸).

## 2. Materials and methods

### 2.1. Protein expression and purification

The coding sequence of the AtSRF6 LRR ectodomain (residues 26–287, UniProt ID A8MQH3; https://uniprot.org) was obtained as a synthetic gene codon-optimized for expression in *Spodoptera frugiperda* (Twist Bioscience) and was introduced by Gibson-assembly cloning (Gibson *et al.*, 2009 ▸) into a modified pFastBac vector (Geneva Biotech), which provides a *Drosophila melanogaster* Bip signal peptide (MKLCILLAVVAFVGLSLD; Soejima *et al.*, 2013 ▸) and a Tobacco etch virus protease (TEV)-cleavable C-terminal StrepII-9×His-tag. For protein expression, *Trichoplusia ni* (strain Tnao38; Hashimoto *et al.*, 2010 ▸) cells were infected with 10 ml of virus in 250 ml of cells at a density of 2.0 × 10^6^ cells ml^−1^, incubated for 24 h at 28°C and 110 rev min^−1^ and then for a further 48 h at 22°C and 110 rev min^−1^. The secreted SRF6 ectodomain was purified from the supernatant by sequential Ni^2+^ (HisTrap Excel; GE Healthcare; equilibrated in 25 m*M* potassium phosphate pH 7.8, 500 m*M* NaCl) and StrepII (Strep-Tactin XT; IBA; equilibrated in 25 m*M* Tris pH 8.0, 250 m*M* NaCl, 1 m*M* EDTA) affinity chromatography, followed by size-exclusion chromatography on a HiLoad 16/600 Superdex 200 pg column (GE Healthcare) equilibrated in 10 m*M* sodium citrate pH 5.0, 250 m*M* NaCl. About 5 mg of purified SRF6 could be obtained from 1 l of insect-cell culture. The monomeric peak fraction was concentrated to 14 mg ml^−1^ using an Amicon Ultra concentrator (molecular-weight cutoff 10 000; Millipore) and directly used for protein crystallization.

The protein samples used to assess the LRR screen were obtained as described: SERK1 (12 mg ml^−1^ in 25 m*M* citric acid pH 5.0, 100 m*M* NaCl; Santiago *et al.*, 2013 ▸), BRI1–BLD–SERK1 (10 mg ml^−1^ in 25 m*M* citric acid pH 5.0, 100 m*M* NaCl; Santiago *et al.*, 2013 ▸), HAESA–IDA (5 mg ml^−1^ in 20 m*M* citric acid pH 5.0, 100 m*M* NaCl; Santiago *et al.*, 2016 ▸), HAESA–IDA–SERK1 (12 mg ml^−1^ in 20 m*M* citric acid pH 5.0, 100 m*M* NaCl; Santiago *et al.*, 2016 ▸), BIR2 (9 mg ml^−1^ in 20 m*M* sodium citrate pH 5.0, 150 m*M* NaCl; Hohmann, Nicolet *et al.*, 2018 ▸), BIR3–SERK1 (14 mg ml^−1^ in 20 m*M* sodium citrate pH 5.0, 150 m*M* NaCl; Hohmann, Nicolet *et al.*, 2018 ▸), PDLP5 (70 mg ml^−1^ in 20 m*M* sodium citrate pH 5.0, 150 m*M* NaCl; Vaattovaara *et al.*, 2019 ▸), SOBIR1 (20 mg ml^−1^ in 20 m*M* sodium citrate pH 5.0, 150 m*M* NaCl; Hohmann & Hothorn, 2019 ▸), GSO1–CIF2 (1 mg ml^−1^ in 20 m*M* sodium citrate pH 5.0, 150 m*M* NaCl; Okuda *et al.*, 2020 ▸), BRI1–A1JME (6 mg ml^−1^ in 20 m*M* citric acid pH 5.0, 250 m*M* NaCl; Caregnato *et al.*, 2025 ▸), BRL3–A1JMF (7 mg ml^−1^ in 20 m*M* citric acid pH 5.0, 250 m*M* NaCl; Caregnato *et al.*, 2025 ▸) and BRL2 (6 mg ml^−1^ in 20 m*M* citric acid pH 5.0, 250 m*M* NaCl; Caregnato *et al.*, 2025 ▸).

For isothermal titration calorimetry (ITC) and grating-coupled interferometry (GCI) assays AtSRF7 (residues 26–287, UniProt ID B5X583) was cloned, expressed and purified as described for SRF6. BRI1, BRL1, BRL3 and SERK3 were expressed and purified as described previously (Hohmann, Santiago *et al.*, 2018 ▸; Caregnato *et al.*, 2025 ▸), as was the expression and purification of BIR1–BIR4 (Hohmann, Nicolet *et al.*, 2018 ▸). For ITC assays, the C-terminal affinity tags in BRL1 and SRF7 were removed by TEV cleavage overnight at 4°C, followed by size-exclusion chromatography into ITC buffer (25 m*M* sodium citrate pH 5.0, 150 m*M* NaCl). BRI1, BRL1 and BRL3 biotinylation for grating-coupled interfero­metry was achieved by incubating the respective Avi-tagged receptor with His-tagged BirA (Fairhead & Howarth, 2015 ▸). The receptor at a final concentration of 20 µ*M* was incubated for 1 h at 30°C with BirA, biotin, ATP and MgCl_2_ at final concentrations of 85 µ*M*, 150 µ*M*, 2 m*M* and 5 m*M*, respectively. The BirA enzyme was subsequently removed by Ni^2+^ affinity chromatography and the biotinylated receptor was concentrated and loaded onto a HiLoad Superdex 200 16/200 pg column (Cytiva) equilibrated in 20 m*M* sodium citrate pH 5.0, 150 m*M* NaCl. The C-terminal affinity tags from SRF6, SRF7, SERK3 and BIR1–BIR4 were removed for these assays as described above.

### 2.2. LRR protein crystallization screening

The LRR crystallization screen was prepared in 96 50 ml Falcon tubes from stock solutions. Precipitants: PEG 1000 [Sigma #81188, 50%(*w*/*v*) stock solution], PEG 3350 [Sigma #88276, 50%(*w*/*v*) stock solution], PEG 8000 [Sigma #89510, 40%(*w*/*v*) stock solution], sodium malonate (3.4 *M* stock solution, pH 4.0–8.0, Hampton Research), ammonium sulfate (Sigma #A4418, 4 *M* stock solution). Salts: ammonium sulfate (Sigma #A4418, 2 *M* stock solution), lithium sulfate (Sigma #13029, 0.5 *M* stock solution), sodium chloride (Sigma #S9888, 2 *M* stock solution), sodium citrate [0.5 *M* stock solution from 0.5 *M* citric acid (#Sigma 251275) with 1.5 *M* sodium hydroxide], ammonium acetate (Sigma #09688, 0.5 *M* stock solution), magnesium chloride (Sigma #M2393, 0.5 *M* stock solution), lithium chloride (Sigma #L9650, 2 *M* stock solution). Buffers: citric acid/NaOH pH 4.0 (Sigma #251275, 1 *M* stock solution), sodium acetate/acetic acid pH 5.5 (Sigma #S8750, 1 *M* stock solution), bis-Tris–HCl pH 7.0 (Sigma B7535, 1 *M* stock solution), Tris base/HCl pH 8.5 (Sigma #T4661, 1 *M* stock solution). Crystallization experiments were performed at room temperature in 96-well MRC 2-well sitting-drop plates (SWISSCI #MRC96T-UVP). Drops were composed of 0.2 µl protein solution and 0.2 µl crystallization buffer suspended over 100 µl of the latter as reservoir solution. The second drop was set up with the protein diluted 1:3 in protein storage buffer. Plates were inspected after 24 h, 3 d and two months on a CX31 microscope (Olympus). In the case of AtSRF6, diffraction-quality crystals appeared after 3 d in LRR screen conditions C5 [25%(*w*/*v*) PEG 3350, 0.2 *M* sodium chloride, 0.1 *M* citric acid pH 4.0] and F9 [20%(*w*/*v*) PEG 8000, 0.2 *M* magnesium chloride, 0.1 *M* citric acid pH 4.0]. A needle-shaped crystal (∼300 × 50 × 50 µm) from condition F9 was transferred to reservoir solution supplemented with 15%(*v*/*v*) glycerol and flash-cooled in liquid N_2_.

### 2.3. Crystallographic data collection, structure solution and refinement

Redundant sulfur single-wavelength anomalous dispersion (SAD) data (λ = 2.079 Å, five 360° wedges at 0.1° oscillation, with χ set to −20°, −10°, 0°, 10°, 20°) were collected to 2.3 Å resolution on beamline X06DA of the Swiss Light Source (SLS), Villigen, Switzerland equipped with a PILATUS 2M-F detector (Dectris) and a multi-axis goniometer. Next, the needle-shaped crystal was translated and an additional high-resolution native dataset (λ = 0.978 Å, one 360° wedge at 0.1° oscillation) was collected to 1.5 Å resolution. Data were processed and scaled with *XDS* and *XSCALE*, respectively (Kabsch, 1993 ▸). Analysis with *phenix.xtriage* (Zwart *et al.*, 2005 ▸) indicated that the anomalous signal of the scaled SAD dataset extended only to ∼4.5 Å. Therefore, the native dataset was input into the *MORDA* automatic molecular-replacement (MR) pipeline (https://www.ccp4.ac.uk/morda-automatic-molecular-replacement-pipeline/), which returned a marginal solution in space group *P*4_3_2_1_2 (translation function *Z*-score 8.4, *R*_work_ = 0.486, *R*_free_ = 0.495) using a single molecule of the previously reported *Arabidopsis* POLLEN RECEPTOR-LIKE KINASE 6 (PRK6) ectodomain structure in the asymmetric unit (PDB entry 5y9w; Zhang *et al.*, 2017 ▸). The *MORDA* solution was input into *Phaser* for MR-SAD phasing against the SAD dataset at 2.3 Å [the starting figure of merit (FOM) was 0.309 at 2.3 Å resolution], yielding four putative sulfur sites by log-likelihood-gradient completion (the final FOM was 0.475). After merging with the high-resolution native dataset, these starting phases were used for automatic model building in *ARP*/*wARP* (Langer *et al.*, 2008 ▸). The resulting structure was completed in iterative cycles of manual model correction in *Coot* (Emsley & Cowtan, 2004 ▸) and restrained refinement in *REFMAC*5 (Murshudov *et al.*, 1997 ▸). The final model had excellent stereochemistry as assessed with *phenix.molprobity* (Davis *et al.*, 2007 ▸). Structural diagrams were prepared with *PyMOL* (https://pymol.org) and *ChimeraX* (Meng *et al.*, 2023 ▸). Phased anomalous difference maps were generated with *phenix.find_peaks_holes* and were displayed in *ChimeraX*.

### 2.4. Analytical size-exclusion chromatography

Analytical size-exclusion chromatography (SEC) experiments were performed on a Superdex 200 Increase 10/300 GL column (GE Healthcare) pre-equilibrated in 20 m*M* sodium citrate pH 5.0, 250 m*M* NaCl. 500 µg of a mixture containing the BRL1 and SRF6 ectodomains in a 1:1 molar ratio was injected in a volume of 100 µl onto the column and elution at 0.75 ml min^−1^ was monitored by ultraviolet absorbance at λ = 280 nm. Peak fractions were analysed by SDS–PAGE.

### 2.5. Isothermal titration calorimetry

The ITC experiment was performed at 25°C using a Nano ITC (TA Instruments) with a 1.0 ml standard cell and a 250 µl titration syringe. SRF6 and BRL1 samples were prepared by gel filtration into ITC buffer (25 m*M* sodium citrate pH 5.0, 150 m*M* NaCl). 10 µl SRF7 aliquots (∼220 µ*M*) were injected into ∼23 µ*M* BRL1 in the cell at 150 s intervals in the absence of a BR. ITC data were corrected for the heat of dilution by subtracting the mixing enthalpies for titrant solution injections into protein-free ITC buffer. Data were analysed using the *NanoAnalyze* program (version 3.5) as provided by the manufacturer.

### 2.6. SRF-family phylogeny

A multiple sequence alignment of the SRF1 (TAIR ID AT2G20850; https://www.arabidopsis.org/), SRF2 (AT5G06820), SRF3 (AT4G03390), SRF5 (AT1G78980), SRF6 (AT1G53730), SRF7 (AT3G14350), SRF8 (AT4G22130) and SRF9/SUB (AT1G11130) protein sequences was generated with *Prob­align* (Roshan & Livesay, 2006 ▸). The phylogenetic tree was generated with *IQ-TREE* 2 (Minh *et al.*, 2020 ▸) and displayed in *FigTree* (https://tree.bio.ed.ac.uk/software/figtree/).

### 2.7. Grating-coupled interferometry

GCI assays were performed on a Creoptix WAVE system (Malvern Panalytical). Binding of the isolated SRF6, SRF7 and SERK3 (positive control) was measured by amine-coupling the BRI1, BRL1, BRL3, BIR1, BIR2, BIR3 or SERK3 ectodomains (ligand) onto 2PCP WAVEchips (quasi-planar polycarboxylate surface; Creoptix AG, Switzerland). Chips were conditioned using borate buffer (100 m*M* sodium borate pH 9.0, 1 *M* NaCl; Xantec, Germany) and the respective ligand was immobilized on the chip surface via a standard amine-coupling protocol, which consisted of 7 min activation with a 1:1 mixture of 400 m*MN*-(3-dimethylaminopropyl)-*N*′-ethylcarbodiimide hydrochloride and 100 m*MN*-hydroxysuccinimide (both from Xantec, Germany), followed by injection of the ligand (1–50 µg ml^−1^) in 10 m*M* sodium acetate pH 5.0 (Sigma, Germany) until the desired density was reached, and quenching with 1 *M* ethanolamine pH 8.0 for 7 min (Xantec, Germany). BSA (0.5% in 10 m*M* sodium acetate pH 5.0; BSA from Roche, Switzerland) was used to passivate the surface between ligand injection and ethanol­amine quenching. The isolated SRF6, SRF7 or SERK3 ectodomains were used as analytes. Kinetic analyses were performed at 25°C with a 1:2 dilution series from 2 µ*M* in 20 m*M* sodium citrate pH 5.0, 250 m*M* NaCl, 100 n*M* brassinolide, with blank injections for double referencing and DMSO calibration for bulk correction. Data correction and analysis was performed with the Creoptix *WAVEcontrol* software (corrections applied: *X* and *Y* offset, DMSO calibration and double referencing). Data were fitted to either one-to-one binding models or mass-transport-limited models using bulk correction.

## 3. Results

### 3.1. A high-throughput crystallization screen for extracellular LRR proteins

The recombinant expression and purification of the first plant LRR-RK ectodomain in baculovirus-infected insect cells yielded only 50–200 µg of purified BRI1 per litre of cell culture (Hothorn *et al.*, 2011 ▸). For the subsequent crystallization of the BRI1–BLD–SERK1 complex (Santiago *et al.*, 2013 ▸), a tailored crystallization screen was therefore developed. The screen simply combined the crystallization conditions reported for the few LRR domain structures that had been previously reported for different animal and plant extracellular proteins (Table 1 ▸; Uff *et al.*, 2002 ▸; He *et al.*, 2003 ▸; Di Matteo *et al.*, 2003 ▸; Kim *et al.*, 2005 ▸; Choe *et al.*, 2005 ▸; Bell *et al.*, 2005 ▸; Liu *et al.*, 2008 ▸; Han *et al.*, 2008 ▸; Hothorn *et al.*, 2011 ▸; She *et al.*, 2011 ▸). The conditions were replicated using a buffer system covering a pH range of 4.0–8.5 (the mean pH of the screen is 5.4; Table 2 ▸). This was based on the idea that extracellular proteins usually reside in an acidic environment (the pH range of the plant apoplast is 4.5–6.5; Almeida & Huber, 1999 ▸) and that the mean pH of many commercially available, high-throughput crystallization screens is close to neutral (Crystal Screen HT, pH 6.6; Index HT, mean pH 6.8; PEG Ion, pH 6.8; Hampton Research).

**Figure 1:**
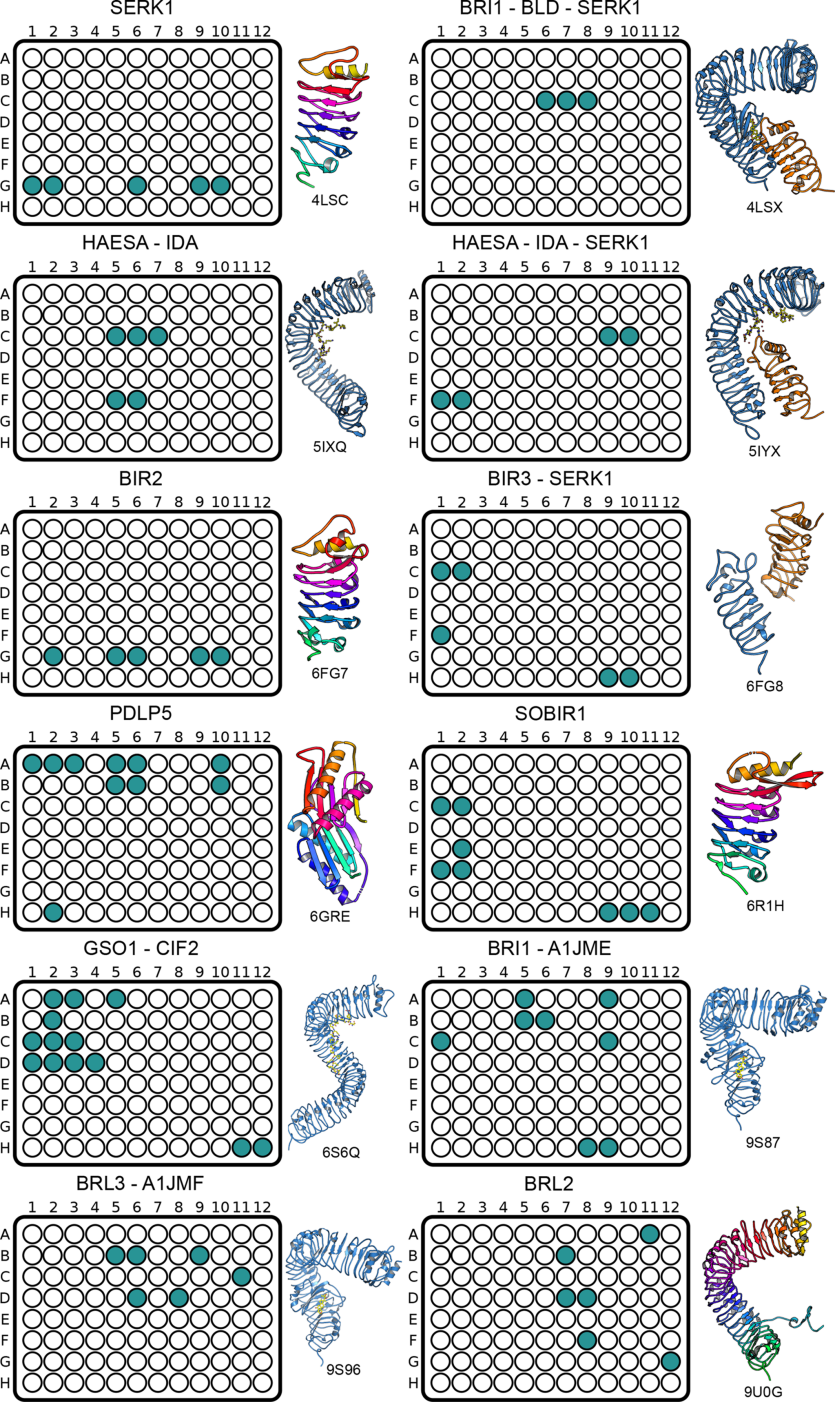
Initial crystallization hits for ten different plant receptor kinases obtained using the LRR crystallization screen. Shown are schematic representations of a 96-well high-throughput crystallization screen plate with positive hits highlighted in cyan, observed after two months of incubation at room temperature. A ribbon diagram of the respective structure is shown alongside, with isolated ectodomain structures coloured from red (N-terminus) to green (C-terminus). Receptor–ligand complexes are shown in blue (ribbon diagram) and yellow (bond representation), respectively. Co-receptor kinases are depicted in orange.

Despite its rather simple design principle, the LRR crystallization screen enabled the crystallization and structural analysis of the steroid receptor kinase BRI1^sud1^, the isolated ectodomain of the co-receptor kinase SERK1 and the BRI1^sud1^–BLD–SERK1 complex (Santiago *et al.*, 2013 ▸; Fig. 1 ▸). Over the years, our laboratory has used the LRR screen to determine the structures of several additional plant receptor kinase domains, including isolated LRR ectodomains (Hohmann, Nicolet *et al.*, 2018 ▸; Hohmann, Santiago *et al.*, 2018 ▸; Hohmann & Hothorn, 2019 ▸), receptor–small molecule and receptor–peptide ligand complexes (Santiago *et al.*, 2016 ▸; Okuda *et al.*, 2020 ▸; Caregnato *et al.*, 2025 ▸), as well as receptor–co-receptor or regulatory complexes (Santiago *et al.*, 2016 ▸; Hohmann, Nicolet *et al.*, 2018 ▸; Fig. 1 ▸). Outside our laboratory, the screen has been used to crystallize the ectodomains of the cell-wall protein LRX4 in complex with its peptide ligand RALF4 (conditions E6 and E7; Moussu *et al.*, 2020 ▸), the LRR-RK HSL1 (condition A1; Roman *et al.*, 2022 ▸) and the related HSL3 (condition E5; Jiménez-Sandoval *et al.*, 2025 ▸). Finally, the LRR screen was used to crystallize the plant receptor-like protein PDLP5, which has a non-LRR tandem-malectin ectodomain (Vaattovaara *et al.*, 2019 ▸; Fig. 1 ▸).

### 3.2. Structure solution of SRF6 crystallized with the LRR screen

**Figure 2:**
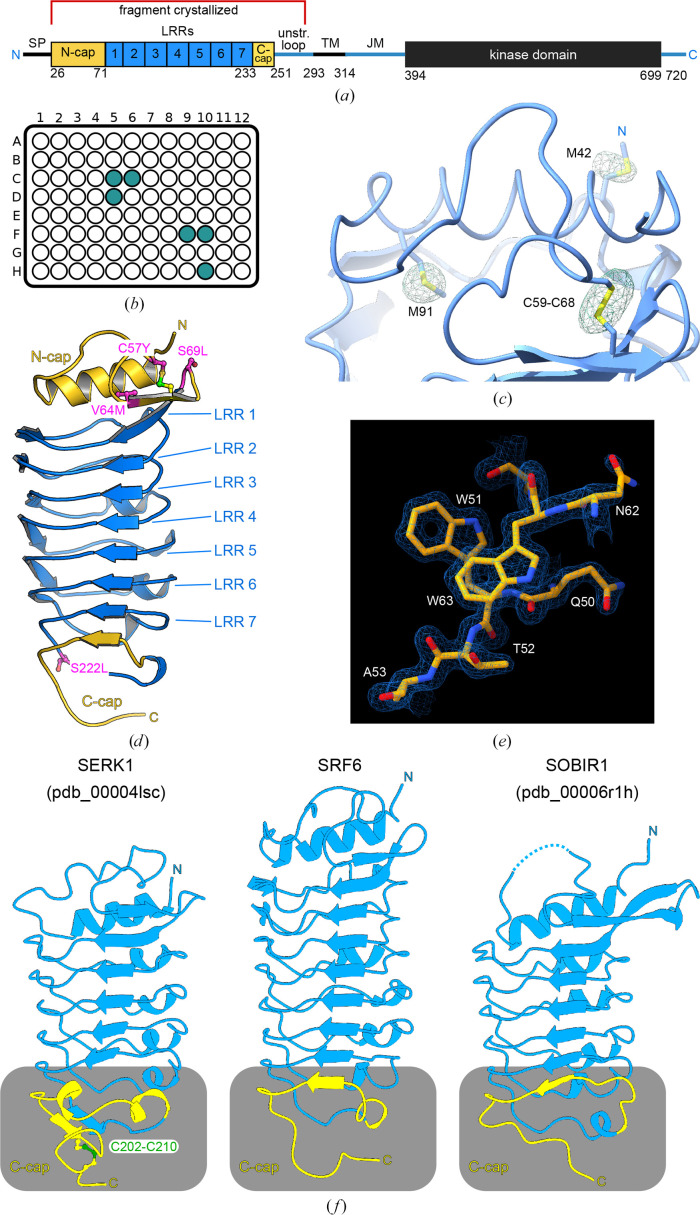
Crystallographic analysis of the LRR ectodomain of the *Arabidopsis *receptor kinase SRF6. (*a*) Schematic representation of SRF6 (SP, signal peptide; N-cap, N-terminal capping domain; C-cap, C-terminal capping domain; TM, transmembrane helix; JM, juxta-membrane motif). The crystallized fragment is indicated by a red line. (*b*) Schematic representation of a 96-well high-throughput crystallization-screen plate; conditions containing SRF6 crystals after 3 d of incubation at room temperature are shown in cyan. (*c*) Ribbon diagram of the SRF6 N-terminal capping domain, with Met42, Cys59, Cys68 and Met91 shown in bond representation and including a phased anomalous difference map contoured at 9σ (green mesh). (*d*) Overall structure of the SRF6 ectodomain. A ribbon diagram is shown; the secondary structure was assigned using *DSSP* (Kabsch & Sander, 1983 ▸). The N- and C-terminal capping domains are shown in yellow and the LRR core in blue. A disulfide bond in the N-terminal capping domain is shown in bond representation; genetic alleles previously characterized for SRF9/SUB are highlighted in magenta. (*e*) Example region of the SRF6 structure covering residues 50–53 and 62–64 (yellow ball-and-stick representation) and including the final (2*F*_o_ − *F*_c_) electron-density map contoured at 1.5σ (blue mesh). (*f*) Structural comparison of the C-terminal capping domains in the *Arabidopsis* receptor kinases SERK1 (PDB entry 4lsc), SRF6 and SOBIR1 (PDB entry 6r1h). The LRR ectodomain (blue) structures of SERK1 (r.m.s.d. of ∼1.5 Å comparing 163 corresponding C^α^ atoms) and SOBIR1 (r.m.s.d. of ∼3.4 Å comparing 133 corresponding C^α^ atoms) were superimposed on the SRF6 LRR domain and presented side-by-side in *ChimeraX*. The C-terminal capping domains are highlighted in yellow.

Next, we used the LRR screen to determine the structure of the isolated LRR ectodomain of the *Arabidopsis thaliana* receptor kinase SRF6 (see Fig. 2 ▸*a*). SRF6 crystallized readily in various conditions in the screen, forming diffraction-quality, needle-shaped crystals in conditions E9 and F6 after three days of incubation at room temperature (see Section 2; Fig. 2 ▸*b*). Redundant SAD and a high-resolution native dataset data were collected from a single crystal. The structure of SRF6 was solved by the MR-SAD method implemented in *Phaser* (McCoy *et al.*, 2007 ▸). The solution contains a single monomer in the asymmetric unit and four sulfur sites corresponding to a disulfide bridge involving Cys59 and Cys88 to Met42 in the N-terminal capping domain and to Met91 in the LRR core (Fig. 2 ▸*c*). The final model was refined against the high-resolution native dataset at 1.50 Å resolution (Table 3 ▸, Fig. 2 ▸*d*). An example region of the final (2*F*_o_ − *F*_c_) map is shown in Fig. 2 ▸(*e*). Overall, SRF6 shares the overall ecto­domain structure with other plant LRR receptor and co-receptor kinases (Hohmann *et al.*, 2017 ▸). The SRF6 LRR domain comprises seven LRRs, rather than six as previously suggested for the related SRF9/SUB (Vaddepalli *et al.*, 2011 ▸l; Fig. 2 ▸*d*). Several genetic missense alleles identified for SRF9/SUB map to the N-terminal capping domain of SRF6, supporting the function of the N-cap in the folding of LRR domains (Truhlar & Komives, 2008 ▸; shown in magenta in Fig. 2 ▸*d*). One of the alleles in SRF9/SUB results in the mutation of Cys57 to tyrosine, and the corresponding cysteine residue in SRF6 is involved in a disulfide bond (Fig. 2 ▸*d*). Functionally, this specific mutation in SRF9/SUB is to the *bri1-5* allele (Cys69-Tyr) in the BR receptor BRI1. It causes BRI1 to be misfolded and retained in the endoplasmic reticulum, triggering the plant’s endoplasmic reticulum-associated degradation (ERAD) pathway (Hong *et al.*, 2009 ▸). In contrast to many other LRR-RK ectodomain structures, SRF6 lacks any visible N-glycans, and its C-terminal capping domain is devoid of a stabilizing disulfide bridge, as previously seen, for example, in the co-receptor kinase SERK1 (Santiago *et al.*, 2013 ▸; Fig. 2 ▸*f*). Instead, the C-terminal cap of SRF6 is structurally reminiscent of the situation described for the immune receptor kinase SOBIR1 (Hohmann & Hothorn, 2019 ▸; Fig. 2 ▸*f*).

### 3.3. Assessing the interaction between the SRF6 and SRF7 ectodomains and BR signalling components

Next, we sought to characterize the potential roles of SRF RKs in BR signalling. Previous studies have reported that SRF6 gene expression is upregulated following BR treatment (Eyüboglu *et al.*, 2007 ▸). In addition, high-throughput inter­action assays revealed that the ectodomains of SRF4, SRF6, SRF7 and SRF9 interacted with the LRR ectodomains of the BR receptors BRI1 and BRL1 in the absence of the steroid ligand (Smakowska-Luzan *et al.*, 2018 ▸). In the same screen, different SRF ectodomains additionally interacted with SERK co-receptor kinases and BIR1–BIR4 receptor pseudokinases (Smakowska-Luzan *et al.*, 2018 ▸). Furthermore, the potato SRF receptor StLRPK1 was reported to constitutively interact with the co-receptor kinase SERK3 in co-immunoprecipitation assays (Wang *et al.*, 2018 ▸).

**Figure 3:**
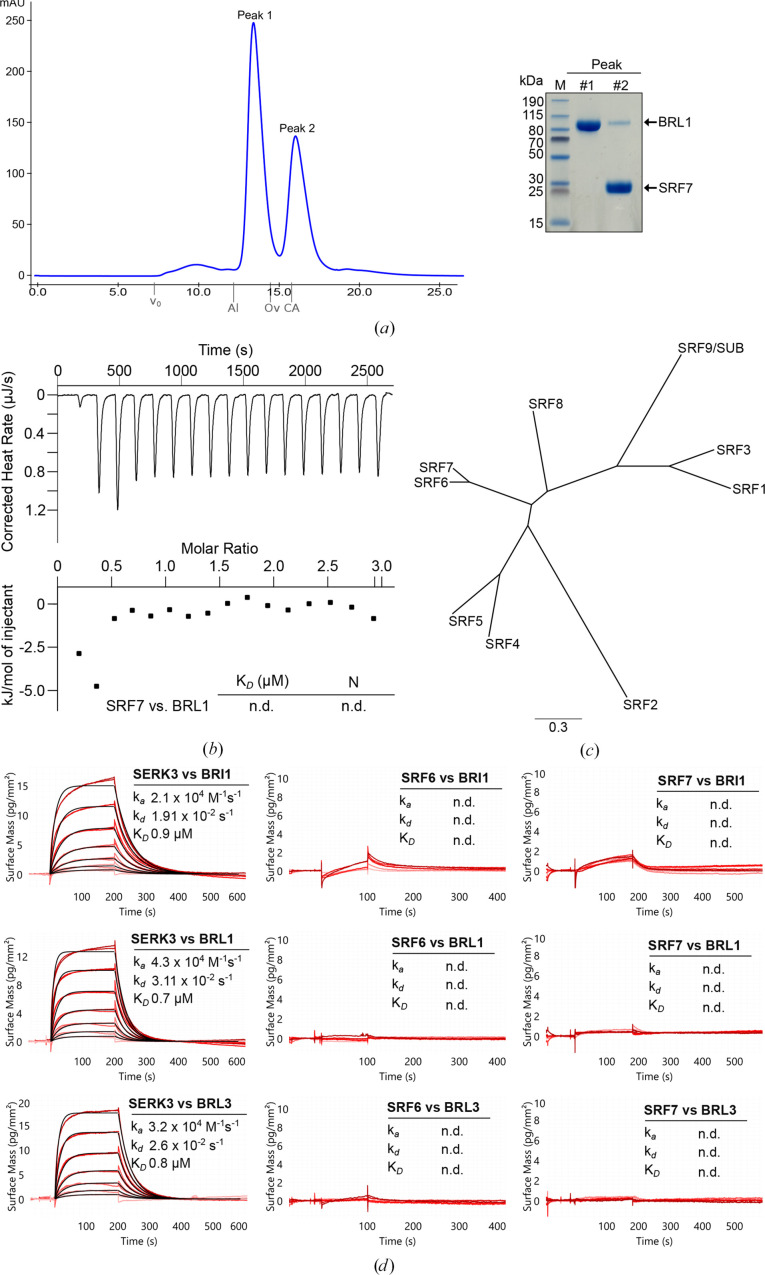
The ectodomains of the SRF6 and SRF7 proteins do not display high-affinity binding to the *Arabidopsis* BR receptors BRI1, BRL1 and BRL3. (*a*) Analytical size-exclusion chromatography of an equimolar mixture of BRL1 and SRF6. The absorbance trace at λ = 280 nm is shown in blue. Indicated are the void volume (*v*_0_) and the elution volumes for molecular-mass standards (Al, aldolase, 158 kDa; Ov, ovalbumin, 43 kDa; CA, carbonic anhydrase, 29 kDa). A Coomassie-stained SDS–PAGE of the peak fractions is shown alongside. (*b*) Isothermal titration calorimetry of SRF6 (in the syringe) versus BRL1 (in the cell). Shown are integrated heat peaks (upper panel) versus time and binding isotherms versus molar ratio of SRF6 ligand (lower panel). (*c*) Phylogenetic tree of the nine SRF family members annotated in the *Arabidopsis* genome. (*d*) Grating-coupled interferometry (GCI) binding kinetics of BRI1, BRL1 and BRL3 versus SRF6 and SRF7 in the presence of 100 n*M* brassinolide. The known BR co-receptor kinase SERK3 served as a positive control. Sensorgrams are shown with raw data in red and their respective fits in black. Binding kinetics were analysed by a 1:1 binding model. Tabular summaries of kinetic parameters are shown alongside (*k*_a_, association rate constant; *k*_d_, dissociation rate constant; *K*_D_, dissociation constant; n.d., no detectable binding).

We purified the LRR ectodomains of SRF6 and SRF7 (see Section 2) and examined their interactions with components of the BR signalling pathway. When mixed in equimolar ratios in the absence of BRs, the BRL1 and SRF7 ectodomains did not co-migrate in analytical size-exclusion chromatography assays (Fig. 3 ▸*a*). Consistently, no interaction was detected by isothermal titration calorimetry in the absence of the steroid ligand brassinolide (Fig. 3 ▸*b*).

As an alternative approach, we analysed the binding of SRF6 and its close homologue SRF7 (Fig. 2 ▸*c*; ∼70% amino-acid sequence identity) by grating-coupled interferometry (GCI). The LRR ectodomains of the BR receptor kinases BRI1, BRL1 and BRL3 bound the co-receptor kinase SERK3 in the presence of brassinolide, consistent with previous reports (Fig. 2 ▸*d*; Hohmann, Santiago *et al.*, 2018 ▸; Caregnato *et al.*, 2025 ▸). In contrast, no binding was detected when either the SRF6 or SRF7 ectodomain was used as the analyte and in the presence of brassinolide (Fig. 3 ▸*d*).

**Figure 4:**
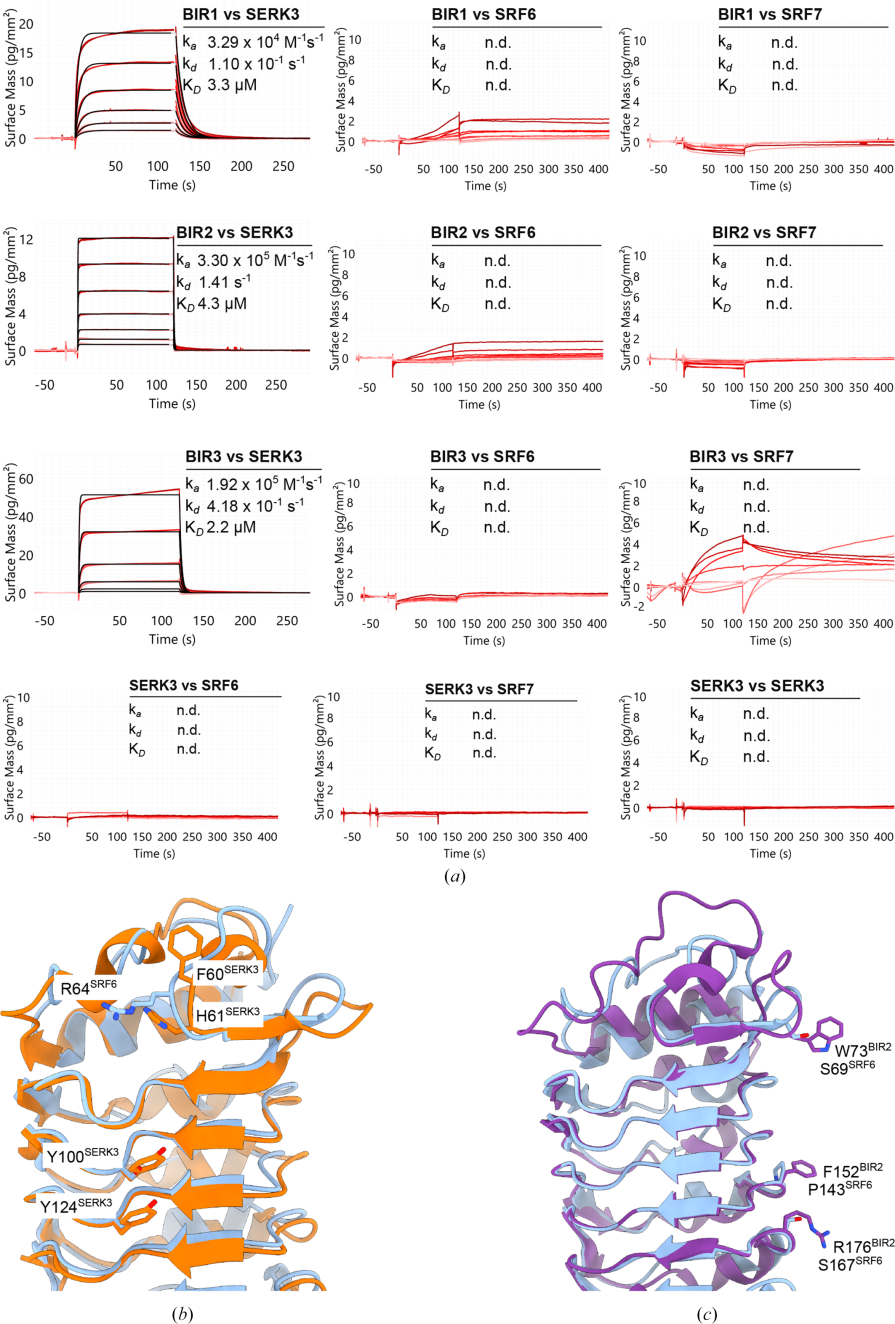
The ectodomains of SRF6 and SRF7 do not display high-affinity binding to *Arabidopsis* BIR receptor pseudokinases or to SERK3. (*a*) Grating-coupled interferometry (GCI) binding kinetics of BIR1, BIR2, BIR3 and SERK3 versus SRF6 and SRF7. The known, ligand-independent interaction between BIRs and SERK3 served as a positive control. Sensorgrams are shown with raw data in red and their respective fits in black. Binding kinetics were analysed by a 1:1 binding model. Tabular summaries of kinetic parameters are shown alongside (*k*_a_, association rate constant; *k*_d_, dissociation rate constant; *K*_D_, dissociation constant; n.d., no detectable binding). (*b*) Key residues required for BR hormone binding and BRI1 receptor association in SERK3 are not conserved in SRF6. A structural superposition of the SRF6 (blue ribbon diagram) and SERK3 (orange, PDB entry 8wec) ectodomains is shown (r.m.s.d. of ∼0.95 Å comparing 175 corresponding C^α^ atoms). Phe60 and His61 in SERK3, which are part of the steroid hormone binding site in the BRI1–SERK complex, correspond to Arg64 and Gly65 in SRF6, respectively. Tyr100 and Tyr124 in SERK3 involved in the binding of the BRI1 LRR domain correspond to Ser104 and Ala126 in SRF6. (*c*) Residues in BIR receptor pseudokinases involved in the interaction with SERK proteins are not conserved in SRF6. A structural superposition of SRF6 and BIR2 (purple, PDB entry 6fg7, r.m.s.d. of ∼0.92 Å comparing 113 corresponding C^α^ atoms) reveals that the distal SERK binding surface in BIR2 involving Trp73, Phe152 and Arg176 is not conserved in SRF6.

BIR receptor pseudo-kinases have been identified as negative regulators of BR signalling (Imkampe *et al.*, 2017 ▸), a function that depends on their LRR domains constitutively interacting with the ectodomains of SERK co-receptor kinases (Ma *et al.*, 2017 ▸; Hohmann, Nicolet *et al.*, 2018 ▸). The LRR domains of BIR1–BIR3, immobilized on the GCI chip (see Section 2), all interacted with the SERK3 ectodomain, as previously reported (Hohmann, Santiago *et al.*, 2018 ▸; Fig. 4 ▸*a*). In contrast, no interaction was detected for SRF6 or SRF7 (Fig. 4 ▸). Similarly, no binding was observed when testing interactions between SRF6 or SRF7 and SERK3 coupled to the chip (Fig. 4 ▸*a*).

Together, our experiments did not reveal direct, high-affinity interactions between the LRR domains of SRF6 or SRF7 and early components of the BR signalling pathway.

## 4. Discussion

Structural biology has provided important insights into the ligand-recognition and receptor-activation mechanisms of plant membrane receptor kinases (Hohmann *et al.*, 2017 ▸; Song *et al.*, 2017 ▸; Moussu & Santiago, 2019 ▸). However, the recombinant expression of these proteins and the production of diffraction-quality crystals remain significant challenges. The LRR crystallization screen described here enabled the successful crystallization and structure determination of several plant RK ectodomains and ectodomain complexes (Fig. 1 ▸). We speculate that the relatively low pH of the screen conditions is a key factor promoting the crystallization of extracellular proteins in otherwise standard PEG- or salt-based crystallization buffers. The individual conditions can be readily prepared from common crystallization stock solutions and stored in sterile-filtered 50 ml Falcon tubes, facilitating the long-term preservation and routine use of this in-house screen.

The SRF6 ectodomain crystallized readily in several LRR screen conditions and its structure could be determined at high resolution by MR-SAD. The resulting model reveals a compact LRR ectodomain architecture that overall resembles other plant LRR receptor kinases and co-receptor kinases, supporting the notion that SRF proteins belong to the structurally conserved LRR-RK superfamily (Fig. 2 ▸).

Motivated by previous reports linking SRF proteins to BR responses, we have examined whether the SRF6 and SRF7 ectodomains interact directly with key components of the BR signalling pathway. However, using multiple complementary biochemical approaches, including analytical size-exclusion chromatography, isothermal titration calorimetry and grating-coupled interferometry, we were unable to detect direct interactions between the ectodomains of SRF6 or SRF7 and BR receptors, co-receptor kinases and receptor pseudo-kinases (Figs. 3 ▸ and 4 ▸). These biochemical observations are consistent with the absence in SRF6 of key residues previously shown to be essential for BR ligand binding (corresponding to Phe60 and His6 in SERK31) and for BR receptor–co-receptor interactions (corresponding to Tyr100 and Tyr124 in SERK3) (Fig. 4 ▸*b*; Santiago *et al.*, 2013 ▸; Hohmann, Santiago *et al.*, 2018 ▸). Likewise, the SERK-binding interface previously identified in the LRR domains of BIR receptor pseudokinases is not conserved in SRF6, providing a structural explanation for the lack of detectable interaction between SRF6 and BIR1–BIR4 (Figs. 4 ▸*a* and 4 ▸*c*; Ma *et al.*, 2017 ▸; Hohmann, Nicolet *et al.*, 2018 ▸).

Taken together, these results suggest that the SRF6 and SRF7 ectodomains do not form stable, high-affinity complexes with early components of the BR receptor complex under the conditions tested. This observation contrasts with previous high-throughput interaction screens (Smakowska-Luzan *et al.*, 2018 ▸) and *in planta* biochemical assays (Wang *et al.*, 2018 ▸). One possible explanation is that such interactions may be transient, indirect or mediated by additional factors present in the cellular context but absent from the *in vitro* binding assays used here. Alternatively, SRF receptors may participate in signalling pathways that intersect with BR responses downstream of receptor activation rather than through direct receptor–receptor interactions at the plasma membrane. Future studies will be required to elucidate the physiological functions of SRF receptors in plant organ development and in cell-wall sensing. The extracellular SRF LRR domain may also function as a ligand-binding module for a cell-wall-derived signalling molecule (Bhasin *et al.*, 2025 ▸), an intriguing possibility that warrants further investigation.

## Supplementary Material

PDB reference: STRUBBELIG-RECEPTOR FAMILY 6 ectodomain from *Arabidopsis thaliana*, 28wb

<p>Raw diffraction images and XDS processing files</p>: https://doi.org/10.5281/zenodo.18745496

## Figures and Tables

**Table 1:** LRR ectodomain crystallization conditions reported between 2002 and 2011

Protein	Precipitant	Salt	Buffer	Reference
GPIbα	2 *M* ammonium sulfate		0.1 *M* Tris–HCl pH 6.5	Uff *et al.* (2002 ▸)
NgR	35% PEG 4000	0.25 *M* sodium chloride	0.1 *M* sodium acetate pH 6.5	He *et al.* (2003 ▸)
PGIP	20–30% PEG 4000	0.2 *M* ammonium acetate	0.1 *M* sodium acetate pH 4.6	Di Matteo *et al.* (2003 ▸)
CD14	1.9 *M* lithium sulfate	0.005 *M* nickel chloride	0.1 *M* HEPES pH 7.5	Kim *et al.* (2005 ▸)
TLR3	23% PEG 2000	0.4 *M* ammonium sulfate		Choe *et al.* (2005 ▸)
TL3	25% PEG 4000	0.2 *M* lithium sulfate	0.1 *M* sodium acetate pH 4.6	Bell *et al.* (2005 ▸)
mTL3	15% PEG 6000, 5% Dextran 5000	1 *M* lithium chloride	0.1 *M* citric acid pH 4.0	Liu *et al.* (2008 ▸)
mTLR3–dsRNA	13% PEG 3350, 5% Dextran 5000	0.2 *M* ammonium citrate pH 5.5		Liu *et al.* (2008 ▸)
VLR–RBC36	2 *M* ammonium sulfate	0.2 *M* sodium/potassium tartrate	0.1 *M* sodium citrate pH 5.6	Han *et al.* (2008 ▸)
BRI1–BLD	14% PEG 4000	0.2 *M* ammonium sulfate	0.1 *M* citric acid pH 4.0	Hothorn *et al.* (2011 ▸)
BRI1–BLD	20% PEG 3350	0.2 *M* ammonium sulfate		She *et al.* (2011 ▸)

**Table 2:** LRR screen formulation

Well	Precipitant	Salt	Buffer
A1	30% PEG 1000	0.2 *M* ammonium sulfate	0.1 *M* citric acid pH 4.0
A2	30% PEG 1000	0.2 *M* ammonium sulfate	0.1 *M* sodium acetate pH 5.5
A3	30% PEG 1000	0.2 *M* ammonium sulfate	0.1 *M* bis-Tris pH 7.0
A4	30% PEG 1000	0.2 *M* ammonium sulfate	0.1 *M* Tris pH 8.5
A5	25% PEG 3350	0.2 *M* ammonium sulfate	0.1 *M* citric acid pH 4.0
A6	25% PEG 3350	0.2 *M* ammonium sulfate	0.1 *M* sodium acetate pH 5.5
A7	25% PEG 3350	0.2 *M* ammonium sulfate	0.1 *M* bis-Tris pH 7.0
A8	25% PEG 3350	0.2 *M* ammonium sulfate	0.1 *M* Tris pH 8.5
A9	20% PEG 8000	0.2 *M* ammonium sulfate	0.1 *M* citric acid pH 4.0
A10	20% PEG 8000	0.2 *M* ammonium sulfate	0.1 *M* sodium acetate pH 5.5
A11	20% PEG 8000	0.2 *M* ammonium sulfate	0.1 *M* bis-Tris pH 7.0
A12	20% PEG 8000	0.2 *M* ammonium sulfate	0.1 *M* Tris pH 8.5
B1	30% PEG 1000	0.2 *M* lithium sulfate	0.1 *M* citric acid pH 4.0
B2	30% PEG 1000	0.2 *M* lithium sulfate	0.1 *M* sodium acetate pH 5.5
B3	30% PEG 1000	0.2 *M* lithium sulfate	0.1 *M* bis-Tris pH 7.0
B4	30% PEG 1000	0.2 *M* lithium sulfate	0.1 *M* Tris pH 8.5
B5	25% PEG 3350	0.2 *M* lithium sulfate	0.1 *M* citric acid pH 4.0
B6	25% PEG 3350	0.2 *M* lithium sulfate	0.1 *M* sodium acetate pH 5.5
B7	25% PEG 3350	0.2 *M* lithium sulfate	0.1 *M* bis-Tris pH 7.0
B8	25% PEG 3350	0.2 *M* lithium sulfate	0.1 *M* Tris pH 8.5
B9	20% PEG 8000	0.2 *M* lithium sulfate	0.1 *M* citric acid pH 4.0
B10	20% PEG 8000	0.2 *M* lithium sulfate	0.1 *M* sodium acetate pH 5.5
B11	20% PEG 8000	0.2 *M* lithium sulfate	0.1 *M* bis-Tris pH 7.0
B12	20% PEG 8000	0.2 *M* lithium sulfate	0.1 *M* Tris pH 8.5
C1	30% PEG 1000	0.2 *M* sodium chloride	0.1 *M* citric acid pH 4.0
C2	30% PEG 1000	0.2 *M* sodium chloride	0.1 *M* sodium acetate pH 5.5
C3	30% PEG 1000	0.2 *M* sodium chloride	0.1 *M* Bis-Tris pH 7.0
C4	30% PEG 1000	0.2 *M* sodium chloride	0.1 *M* Tris pH 8.5
C5	25% PEG 3350	0.2 *M* sodium chloride	0.1 *M* citric acid pH 4.0
C6	25% PEG 3350	0.2 *M* sodium chloride	0.1 *M* sodium acetate pH 5.5
C7	25% PEG 3350	0.2 *M* sodium chloride	0.1 *M* bis-Tris pH 7.0
C8	25% PEG 3350	0.2 *M* sodium chloride	0.1 *M* Tris pH 8.5
C9	20% PEG 8000	0.2 *M* sodium chloride	0.1 *M* citric acid pH 4.0
C10	20% PEG 8000	0.2 *M* sodium chloride	0.1 *M* sodium acetate pH 5.5
C11	20% PEG 8000	0.2 *M* sodium chloride	0.1 *M* bis-Tris pH 7.0
C12	20% PEG 8000	0.2 *M* sodium chloride	0.1 *M* Tris pH 8.5
D1	30% PEG 1000	0.2 *M* sodium citrate	0.1 *M* citric acid pH 4.0
D2	30% PEG 1000	0.2 *M* sodium citrate	0.1 *M* sodium acetate pH 5.5
D3	30% PEG 1000	0.2 *M* sodium citrate	0.1 *M* bis-Tris pH 7.0
D4	30% PEG 1000	0.2 *M* sodium citrate	0.1 *M* Tris pH 8.5
D5	25% PEG 3350	0.2 *M* sodium citrate	0.1 *M* citric acid pH 4.0
D6	25% PEG 3350	0.2 *M* sodium citrate	0.1 *M* sodium acetate pH 5.5
D7	25% PEG 3350	0.2 *M* sodium citrate	0.1 *M* bis-Tris pH 7.0
D8	25% PEG 3350	0.2 *M* sodium citrate	0.1 *M* Tris pH 8.5
D9	20% PEG 8000	0.2 *M* sodium citrate	0.1 *M* citric acid pH 4.0
D10	20% PEG 8000	0.2 *M* sodium citrate	0.1 *M* sodium acetate pH 5.5
D11	20% PEG 8000	0.2 *M* sodium citrate	0.1 *M* bis-Tris pH 7.0
D12	20% PEG 8000	0.2 *M* sodium citrate	0.1 *M* Tris pH 8.5
E1	30% PEG 1000	0.2 *M* ammonium acetate	0.1 *M* citric acid pH 4.0
E2	30% PEG 1000	0.2 *M* ammonium acetate	0.1 *M* sodium acetate pH 5.5
E3	30% PEG 1000	0.2 *M* ammonium acetate	0.1 *M* bis-Tris pH 7.0
E4	30% PEG 1000	0.2 *M* ammonium acetate	0.1 *M* Tris pH 8.5
E5	25% PEG 3350	0.2 *M* ammonium acetate	0.1 *M* citric acid pH 4.0
E6	25% PEG 3350	0.2 *M* ammonium acetate	0.1 *M* sodium acetate pH 5.5
E7	25% PEG 3350	0.2 *M* ammonium acetate	0.1 *M* bis-Tris pH 7.0
E8	25% PEG 3350	0.2 *M* ammonium acetate	0.1 *M* Tris pH 8.5
E9	20% PEG 8000	0.2 *M* ammonium acetate	0.1 *M* citric acid pH 4.0
E10	20% PEG 8000	0.2 *M* ammonium acetate	0.1 *M* sodium acetate pH 5.5
E11	20% PEG 8000	0.2 *M* ammonium acetate	0.1 *M* bis-Tris pH 7.0
E12	20% PEG 8000	0.2 *M* ammonium acetate	0.1 *M* Tris pH 8.5
F1	30% PEG 1000	0.2 *M* magnesium chloride	0.1 *M* citric acid pH 4.0
F2	30% PEG 1000	0.2 *M* magnesium chloride	0.1 *M* sodium acetate pH 5.5
F3	30% PEG 1000	0.2 *M* magnesium chloride	0.1 *M* bis-Tris pH 7.0
F4	30% PEG 1000	0.2 *M* magnesium chloride	0.1 *M* Tris pH 8.5
F5	25% PEG 3350	0.2 *M* magnesium chloride	0.1 *M* citric acid pH 4.0
F6	25% PEG 3350	0.2 *M* magnesium chloride	0.1 *M* sodium acetate pH 5.5
F7	25% PEG 3350	0.2 *M* magnesium chloride	0.1 *M* bis-Tris pH 7.0
F8	25% PEG 3350	0.2 *M* magnesium chloride	0.1 *M* Tris pH 8.5
F9	20% PEG 8000	0.2 *M* magnesium chloride	0.1 *M* citric acid pH 4.0
F10	20% PEG 8000	0.2 *M* magnesium chloride	0.1 *M* sodium acetate pH 5.5
F11	20% PEG 8000	0.2 *M* magnesium chloride	0.1 *M* bis-Tris pH 7.0
F12	20% PEG 8000	0.2 *M* magnesium chloride	0.1 *M* Tris pH 8.5
G1	3.4 *M* sodium malonate pH 4.0		
G2	3.4 *M* sodium malonate pH 5.0		
G3	3.4 *M* sodium malonate pH 6.0		
G4	3.4 *M* sodium malonate pH 8.0		
G5	2.5 *M* sodium malonate pH 4.0		
G6	2.5 *M* sodium malonate pH 5.0		
G7	2.5 *M* sodium malonate pH 6.0		
G8	2.5 *M* sodium malonate pH 8.0		
G9	1.8 *M* sodium malonate pH 4.0		
G10	1.8 *M* sodium malonate pH 5.0		
G11	1.8 *M* sodium malonate pH 6.0		
G12	1.8 *M* sodium malonate pH 8.0		
H1	3.5 *M* ammonium sulfate		0.1 *M* citric acid pH 4.0
H2	3.5 *M* ammonium sulfate		0.1 *M* sodium acetate pH 5.5
H3	3.5 *M* ammonium sulfate		0.1 *M* bis-Tris pH 7.0
H4	3.5 *M* ammonium sulfate		0.1 *M* Tris pH 8.5
H5	3.5 *M* ammonium sulfate		0.1 *M* citric acid pH 4.0
H6	3.5 *M* ammonium sulfate		0.1 *M* sodium acetate pH 5.5
H7	3.5 *M* ammonium sulfate		0.1 *M* bis-Tris pH 7.0
H8	3.5 *M* ammonium sulfate		0.1 *M* Tris pH 8.5
H9	20% PEG 3350	1.0 *M* lithium chloride	0.1 *M* citric acid pH 4.0
H10	20% PEG 3350	1.0 *M* lithium chloride	0.1 *M* sodium acetate pH 5.5
H11	20% PEG 3350	1.0 *M* lithium chloride	0.1 *M* bis-Tris pH 7.0
H12	20% PEG 3350	1.0 *M* lithium chloride	0.1 *M* Tris pH 8.5

**Table 3:** SRF6 crystallographic data-collection, phasing and refinement statistics Values in parentheses are for the highest resolution shell.

	Sulfur MR-SAD	High resolution
Data collection		
Wavelength (Å)	2.0785	0.9779
Space group	*P*4_3_2_1_2	*P*4_3_2_1_2
*a*, *b*, *c* (Å)	76.76, 76.76, 86.51	76.72, 76.72, 86.43
α, β, γ (°)	90, 90, 90	90, 90, 90
Resolution (Å)	45.98–2.30 (2.36–2.30)	45.95–1.50 (1.59–1.50)
*R*_meas_†	0.152 (0.872)	0.174 (2.58)
CC_1/2_† (%)	100 (92.5)	100 (50.8)
〈*I*/σ(*I*)〉†	35.0 (4.2)	13.9 (1.1)
Completeness† (%)	99.7 (96.6)	99.9 (99.3)
Multiplicity†	61.8 (25.8)	13.6 (12.9)
Wilson *B* factor† (Å^2^)	22.5	16.7
Phasing		
Resolution (Å)	45.98–2.30	
No. of sites	4	
FOM‡	0.475	
Refinement		
Resolution (Å)		45.95–1.50 (1.54–1.50)
No. of reflections		39715 (2853)
*R*_work_/*R*_free_§		0.162/0.195 (0.319/0.329)
No. of atoms		
Protein		1756
Buffer		15
Water		237
Residue *B* factors§ (Å^2^)		
Protein		19.9
Buffer		30.36
Water		31.45
R.m.s deviations¶		
Bond lengths (Å)		1.82
Bond angles (°)		0.012
*MolProbity* results		
Ramachandran outliers (%)		0
Ramachandran favoured (%)		91.52
*MolProbity* score		1.54
PDB code		28wb

† As defined in *XDS*.

‡ Final figure of merit of the MR-SAD experiment as defined in *Phaser*.

§ As defined in *REFMAC*5.

¶ As defined in *phenix.molprobity*.

## Data Availability

Raw diffraction images and *XDS* processing files have been deposited at https://doi.org/10.5281/zenodo.18745496.
